# Role of Th17 Cells in the Pathogenesis of Human IBD

**DOI:** 10.1155/2014/928461

**Published:** 2014-03-25

**Authors:** Julio Gálvez

**Affiliations:** CIBER-EHD, Department of Pharmacology, Center for Biomedical Research (CIBM), University of Granada, Avenida del Conocimiento s/n, Granada, 18100 Armilla, Spain

## Abstract

The gastrointestinal tract plays a central role in immune system, being able to mount efficient immune responses against pathogens, keeping the homeostasis of the human gut. However, conditions like Crohn's disease (CD) or ulcerative colitis (UC), the main forms of inflammatory bowel diseases (IBD), are related to an excessive and uncontrolled immune response against normal microbiota, through the activation of CD4^+^ T helper (Th) cells. Classically, IBD was thought to be primarily mediated by Th1 cells in CD or Th2 cells in UC, but it is now known that Th17 cells and their related cytokines are crucial mediators in both conditions. Th17 cells massively infiltrate the inflamed intestine of IBD patients, where they produce interleukin- (IL-) 17A and other cytokines, triggering and amplifying the inflammatory process. However, these cells show functional plasticity, and they can be converted into either IFN-**γ** producing Th1 cells or regulatory T cells. This review will summarize the current knowledge regarding the regulation and functional role of Th17 cells in the gut. Deeper insights into their plasticity in inflammatory conditions will contribute to advancing our understanding of the mechanisms that regulate mucosal homeostasis and inflammation in the gut, promoting the design of novel therapeutic approaches for IBD.

## 1. Introduction

**Figure 1:**
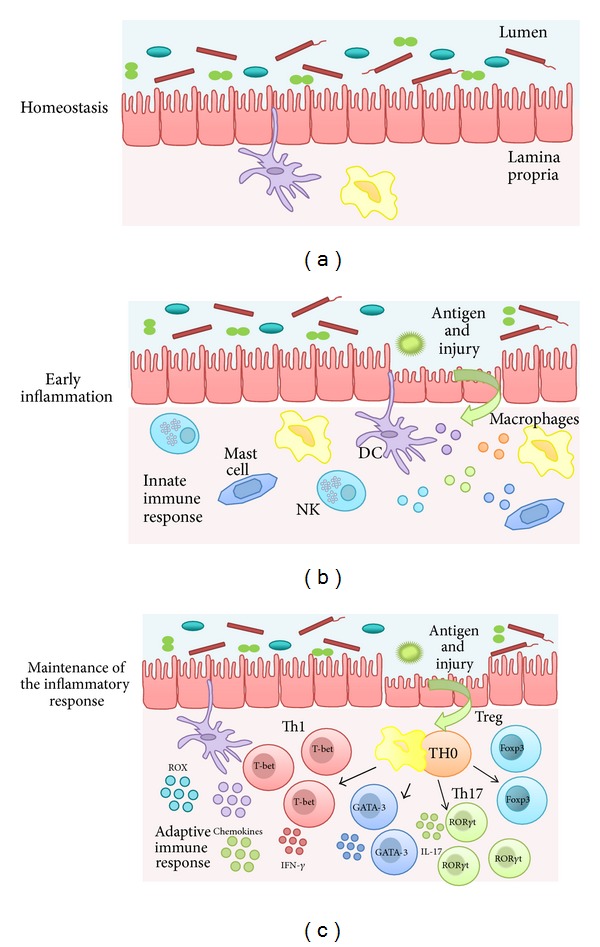
Physiopathology of IBD. (a) The intestine comprises the major single epithelial interface in the body, which is populated by the greatest number and diversity of resident microbes. The intestinal immune system therefore encounters more antigens than any other part of the body and it must discriminate between invasive organisms and harmless antigens, such as food, proteins, and commensal bacteria. The intestinal homeostasis depends on the dynamic crosstalk between the microbiota, the intestinal epithelial cells, and the resident immune cells. (b) Several mechanisms are involved in the regulation of the intestinal homeostasis. The breakdown of this balance triggers the chronic inflammatory process found in inflammatory bowel disease. During early inflammation, foreign antigens activate the different innate immune cells located in the intestine, including natural killer cells, mast cells, neutrophils, macrophages, and dendritic cells. (c) A maintained inflammatory reaction promotes the activation of the adaptive immune response. Abnormally activated effector CD4^+^ T helper (Th) cells synthetize and release different inflammatory mediators that generate the vicious circle of inflammation that leads to chronic tissue injury and epithelial damage.

Inflammatory bowel disease (IBD) is a chronic relapsing inflammatory disorder of the gastrointestinal tract that comprises two major conditions: Crohn's disease (CD) and ulcerative colitis (UC). These pathologies are characterized by abdominal pain, fever, chronic diarrhea, and rectal bleeding due to ulceration of the inner lining of the colon and/or rectum, which can be accompanied by complications such as fistulation, stenosis, and abscesses in CD and megacolon in UC. Acute flares severely impair patient's ability to lead a normal life, frequently requiring hospitalization and surgery, and may even be life threatening. At present, the pathogenesis of IBD remains elusive; however, the altered and chronic activation of the immune and inflammatory cascade in genetically susceptible individuals against unknown components of the luminal microflora seems to play a key role [1, 2] (Figure 1). The intestinal immune system is the largest and most complex component of the immune system in the human being. As the intestine comprises the major single epithelial interface in the body, which is populated by the greatest number and diversity of resident microbes, the intestinal immune system encounters therefore more antigens than any other part of the body and it must discriminate between invasive organisms and harmless antigens, such as food proteins and commensal bacteria [3]. At the same time, strong immune responses are required to protect this physiologically essential tissue, and, in consequence, a fine tuning in the immune responses to luminal antigens is essential to maintain homeostasis.

As expected in any inflammatory process, during the initial inflammatory response that takes place in IBD, the different innate immune cells located in the intestine, including natural killer cells, mast cells, neutrophils, macrophages, and dendritic cells, must be activated by foreign antigens, which at present remain unknown. Afterwards, the maintenance of the inflammatory response with time is promoted through the stimulation of the adaptative immune response, mainly mediated by abnormally activated effector CD4^+^ T helper (Th) cells, in concordance with the reduced rates of apoptosis that have been reported to occur in these cells in human IBD [4]. As a result of this cell activity, there is an upregulation of the synthesis and release of different proinflammatory mediators including reactive oxygen and nitrogen metabolites, eicosanoids, chemokines, and cytokines, which actively contribute to the pathogenic cascade that initiates and perpetuates the intestine inflammatory response in these conditions [5].

When considering effector CD4^+^ Th cells, these typically show functional heterogeneity associated with a different profile of cytokine production, which correlates with the nature of the offending microorganism. The differentiation in specific T-cell types significantly depends on the interactions of specific cytokines with signal transducer and activator of transcription (STAT) factors. The cytokines that promote Th cell polarization are mostly derived from innate immune cells that recognize microbe-associated molecular patterns (MAMPs) in an attempt to establish proinflammatory antimicrobial responses. Initially, when considering immunological functions, transcription factor expression, and cytokine secretion, activated Th cells were divided into two different subsets: Th1 and Th2. Th1 cells secrete interferon-*γ* (IFN-*γ*), a potent activator of intracellular killing by macrophages, with the main role of protecting the host against intracellular pathogens, like some bacteria and viruses, that are capable to survive or replicate within macrophages. Th1 cell development requires the sequential actions of STAT1 and STAT4, induced by IFN-*γ* and interleukin-12 (IL-12), respectively, which promote an enhanced expression of the T-box transcription factor normally expressed in T cells, T-bet. However, Th2 cells express the transcription factor GATA binding protein 3 (GATA-3) and secrete IL-4, IL-5, and IL-13, thus collaborating to host defence against helminths. The existence of a reciprocal regulation of Th1 and Th2 polarization is well known, achieved through the production of their main corresponding cytokines, IFN-*γ* and IL-4, thus enforcing their own expression while inhibiting alternative commitment [6]. Similarly, the related Th1- and Th2-specific transcription factors participate in this process, since T-bet activation can suppress GATA-3 expression [7]. Nevertheless, in addition to their protective functions against invading pathogens, each subtype of Th cells also participates in the development of different immune-related human conditions. In fact, considering human IBD, CD has been traditionally linked with Th1 cells [8], with a predominance of Th1-related cytokines such as IL-12 and IFN*γ* in the mucosa, while UC is characterized by an increased production of IL-5 and IL-13, being therefore linked with Th2 cells [9].

Nevertheless, and more recently, a new subset of Th cells capable of producing IL-17 has been reported to play a key pathogenic role in chronic inflammatory conditions, including IBD [10]. The first reports suggesting the existence of these cells were published in 1999, in studies performed in patients with rheumatoid arthritis and cutaneous inflammation [11, 12]. However, it was six years later when Langrish et al. [13] first described “Th17 cells” as a new subtype of effector Th cells. They were further characterized by the expression of the transcription factor retinoic acid orphan receptor (ROR)*γ*t, but not T-bet or GATA-3 [14], and by their ability to selectively produce high levels of IL-17A and IL-17F, crucial cytokines for host defence against extracellular pathogens. A prominent feature in these cells is a functional plasticity towards the Th1 subset, being reported that some Th17 cells are able to produce both IL-17 and IFN-*γ* [15].

The aim of this review is to summarize the current knowledge regarding the regulation and functional role of Th17 cells, in the context of IBD, focusing on their novel features such as their plasticity and their relationship with regulatory T (Treg) cells, as well as on their heterogeneity in the inflammatory microenvironment of the intestine.

## 2. Th17 Cell Differentiation in the Gut: Role in Intestine Inflammatory Conditions

**Figure 2:**
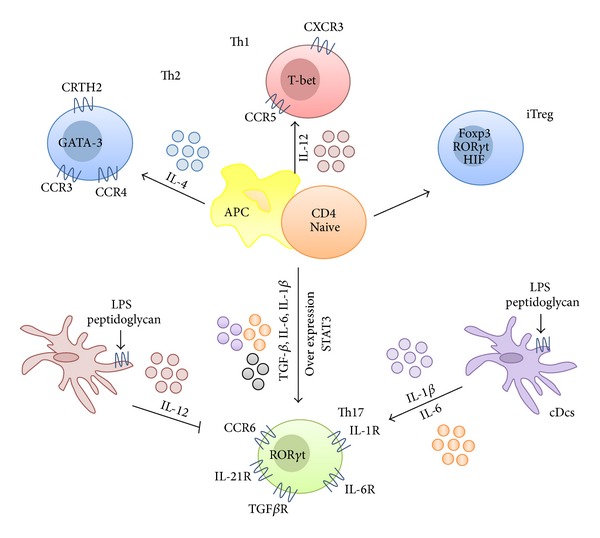
T-cell differentiation into Th17 cells. Naive CD4^+^ T cells, upon contact with antigen-presenting cells (APCs), have the potential to differentiate into Th1, Th2, Th17, and induced regulatory T cells (iTreg), a process controlled by the effector cytokines produced by APCs. Th17 cells are originated from a small subset of naive T cells that express CD161 and cytokine receptors IL-6R, TGF*β*R, IL-23R, IL-21R, and IL-1R, being their polarization into Th17 cells promoted by the combined activity of these cytokines. These cytokines mediate their effects by promoting or repressing the expression of different transcription factors, in particular STAT3 and ROR*γ*t, which are critical for Th17 differentiation and development. It is of great interest to discern the nature of the APCs capable of inducing Th17 responses and the corresponding stimuli involved in this. Monocytes and circulating conventional DCs (cDCs) that produce large amounts of IL-1*β* and IL-6 when activated by lipopolysaccharide (LPS) and peptidoglycan are known to be the most efficient APCs for Th17 differentiation, whereas monocyte-derived DCs that produce IL-12, but not IL-1*β*, in response to LPS or peptidoglycan fail to promote the differentiation of Th17 cells.

Th17 cells have been proposed to be exclusively originated from a small subset of naive T cells expressing the lectin receptor CD161 (CD161^+^CD4^+^ T cell progenitors) [16]. It is well known that Th cell development begins in the thymus; however, the functional differentiation of Th cells in the intestinal mucosa occurs when the T cell encounters an activated antigen-presenting cell (APC), such as dendritic cells (DCs). Naive CD4^+^ T cells have the potential to differentiate into Th1, Th2, induced regulatory T (iTreg) cells, and Th17 cells, being this process driven by the specific effector cytokines produced by APCs (Figure 2). It has been reported that monocytes and circulating conventional DCs (cDCs) activated by LPS and peptidoglycan, that are able to produce large amounts of IL-1*β* and IL-6 but little IL-12, are the most efficient inducers of Th17 differentiation; on the contrary, monocyte-derived DCs that produce IL-12, but not IL-1*β*, when activated by LPS or peptidoglycan, do not promote the differentiation of Th17 cells [17]. It is well known that the cytokines released by APCs activate the Jak-STAT pathway to exert their biological actions; in this regard, the transcription factor STAT3 is crucial for Th17 differentiation when activated by both IL-6 and IL-23 [18]. Supporting this, it has been reported that STAT3 overexpression promoted Th17 development, whereas this process was greatly impaired in mice with STAT3-deficient T cells [19]. Furthermore, STAT3 has also been reported to regulate the expression of the transcription factor ROR*γ*t, which is critical for Th17 differentiation and development; in fact, an impaired ROR*γ*t expression leads in turn to increased expression of other transcription factors such as T-bet and Forkhead box P3 (Foxp3) [19], promoting the development of other Th lineages rather than Th17 cells.

Both constitutive and inducible/inflammatory chemokines actively participate in the migration of both effector and memory T cells to specific intestine tissue sites to exert their corresponding biological function [20]. Therefore, chemokine receptors are differentially expressed on the different effector T-cell subsets: CXCR3 and CCR5 are preferentially expressed on Th1 cells, and CCR3, CCR4 and CRTH2 are preferentially expressed on Th2 cells [21], whereas CCR6 expression has been associated with Th17-cell differentiation [17]. In fact, it has been shown that the specific transcription factors for these lineages, T-bet for Th1 cells, GATA-3 for Th2 cells, and ROR*γ*t for Th17 cells, respectively, control CXCR3, CCR3, and CCR6 genes' transcription in a direct fashion [15].

In addition to chemokine receptors, the Th17 cell progenitors, CD161^+^CD4^+^ T cells, also express several cytokine receptors, including IL-6 receptor (IL-6R), transforming growth factor-beta (TGF)*β* receptor, IL-23R, IL-21R, and IL-1R. Of note, it has been proposed that when these cells are found within a proinflammatory environment in the intestine, characterised by the presence of high levels of IL-6, IL-1*β*, and IL-23, their polarization into Th17 cells is induced [17, 22], as it can occur in intestine inflammation. However, not all these cytokines play the same role in this process. Whereas IL-6 can be considered as an essential cytokine for Th17 differentiation of naive T cells [23], the proinflammatory cytokines IL-1*β* and tumor necrosis factor-alpha (TNF-*α*) assist in the efficiency of this process [24]. The role of IL-23 has not been clearly defined yet: although it was initially reported to be required for Th17 differentiation [25], subsequent studies have revealed that this cytokine might be essential for IL-17-mediated effector function and Th17 cell survival, but not for their differentiation [26].

The controversial role of TGF*β* in the differentiation of human Th17 cells is interesting; while in some studies its presence in the culture media was found indispensable for the induction of ROR*γ*t expression and in turn Th17 differentiation* in vitro* [27], other studies have not stated this requirement [17]. It was then proposed that the role of TGF*β* in Th17 differentiation is dose dependent: while low doses of this cytokine are essential for Th17 differentiation, at high concentrations it inhibits the expression and functions of ROR*γ*t [28, 29]. Additionally, TGF*β* can also promote human Th17 differentiation indirectly due to its ability to suppress T-bet expression and the generation of Th1 cells [30], or by blocking expression of the transcription factors signal STAT4 and GATA-3, thus preventing Th1 and Th2 cell differentiation [31].

### 2.1. Th17 Cell Function

Mature Th17 cells are able to produce several effector molecules, including the cytokines IL-17A and IL-17F, IL-21, IL-22, IL-26, IL-8, IL-10, TNF-*α*, and granulocyte-macrophage colony-stimulating factor (GM-CSF), as well as the chemokine CXC ligands CXCL8 and CCL20. IL-17A is the prototype member of the IL-17 family that is composed by six cytokines: IL-17A, IL-17B, IL-17C, IL-17D, IL-17E, and IL-17F [32]. Both IL-17A and IL-17F have similar functions by targeting both immune and nonimmune cells, and they have been reported to play a key role in the Th17-mediated inflammatory response [33], through the IL-17 receptor A (IL-17RA)-IL-17RC heterodimer [34], showing a much higher affinity for IL-17A than for IL-17F. The binding of IL-17 to its receptor induces the recruitment of the adaptor protein ACT1, which in turn stimulates TNF receptor-associated factor 6 to activate the NF-*κ*B and MAPK pathways [32]. Both IL-17A and IL-17F play a key role in the recruitment, activation, and migration of granulocytes, being also able to target other cells, including epithelial cells, endothelial cells, fibroblasts, and macrophages, to induce proinflammatory mediators like IL-6, TNF-*α*, IL-1*β*, GM-CSF, G-CSF, PGE_2_, nitric oxide and matrix metalloproteinases, and different chemokines, such as CXCLI, CXCL8, CCL2, CCL7, and CCL20 [32, 35]. CCL20 has antimicrobial and chemoattractive properties and it is a ligand for the chemokine CC receptor CCR6, which is expressed by dendritic cells, B cells, and different subsets of T cells. Of note, Th17 cells also express CCR6, and the release of CCL20 in response to IL-17 promotes further attraction and recruitment of Th17 cells to the inflammatory site [36], indicating that CCL20 may have an important role in sustaining Th17-mediated effects in inflammation.

Regarding the other cytokines produced by Th17 cells, IL-21 is a member of the IL-2 family of cytokines and exerts its function via the IL-21 receptor, which is expressed in different myeloid cells, as well as in B and T cells [32]. Among the biological effects of IL-21, it has been reported that this cytokine promotes Th1 responses [37] and also potently induces Th17 differentiation from CD4^+^ T cells in an autocrine way, amplifying Th17 responses and therefore inducing its own expression in an autocrine loop [38, 39]. IL-22 belongs to the IL-10 family of cytokines, and it is produced by mature Th17 cells through IL-23-mediated STAT3 activation [32]. The binding of IL-22 to its corresponding receptor (IL-22R), which is highly expressed in epithelial cells and parenchymal tissues, but not on immune cells, activates the STAT3 and MAPK pathways and promotes the production and release of antimicrobial agents and *β*-defensins, being therefore considered an essential cytokine for the immune barrier function of epithelial cells [40, 41]. Finally, the production of either TNF-*α* or GM-CSF by Th17 cells contributes to the activation, survival, and recruitment of neutrophils in different inflammatory conditions, including IBD [42].

### 2.2. Th17 Cells in Intestine Inflammation

Nowadays, it is well known that the inflamed gastrointestinal mucosa of patients with IBD has a massive infiltration of Th17 cells and that Th17-related cytokines are produced in excess in both CD and UC tissues [43], thus proposing a key role for these cells in these intestine conditions [44]. Fujino et al. [45] showed that there are an increased number of IL-17-producing cells in the inflamed gut of patients with IBD in comparison to healthy controls. In line with this, it has been shown that RNA transcripts for IL-17A and IL-17F are upregulated in the inflamed mucosa of IBD patients [45, 46]. Supporting these observations, a correlation between disease severity and levels of IL-17 secreted by PBMCs from UC patients has also been observed [47]. In addition to IL-17A and IL-17F, various studies have reported an increased production of other Th17 cytokines in the inflamed gut of IBD patients, such as IL-22 and IL-26 [48], or IL-21 and IL-23, which in turn exacerbate inflammation by promoting Th1 and Th17 responses [49]. Moreover, IBD genomewide association studies and candidate gene studies have shown that several polymorphisms in Th17-related genes, such as STAT3 or IL-23R, are associated with IBD, thus supporting the involvement of the Th17 pathway in its IBD pathogenesis [50].

Based on this, it is obvious that Th17 cells play an important role in IBD pathogenesis, which was solely attributed to Th1 and Th2 cells in the past. Different studies have proposed a singular prominent role for each Th cell subset in either CD or UC. In this sense, it has been shown that the numbers of IFN-*γ* secreting T cells in peripheral blood from IBD patients significantly correlated with disease severity in CD but not in UC [47]. Other laboratories have reported that, in response to IL-23, IL-17 production by intestinal lymphocytes was only significantly enhanced in UC, while in CD, IFN-*γ* production was induced instead [51]. These observations might indicate that IFN-*γ* mediates Th1 inflammation in CD while IL-17 mediates disease in UC or that the capacity to produce IL-17 by Th17 cells may be disturbed by the enhanced Th1 cytokines present in CD intestinal mucosa [51]. Furthermore, elevated levels of CCL20, a Th17 cell chemoattractant [52], have also been documented in IBD mucosa, where CCL20 production seems to be positively regulated by IL-21 [53]. IL-21 is overproduced in the intestine of IBD patients, but the vast majority of IL-21-producing CD4^+^ T cells coexpress IFN-*γ* and not IL-17A, thus suggesting that Th1, and not Th17, cells are the major sources of IL-21 in the human gut [54].

However, although a pathogenic role in intestine inflammation has been ascribed to Th17 cells, different studies in experimental colitis have reported tissue-protective effects of Th17-type cytokines in the gut. In fact, the neutralization of IL-17A, either by antibody treatment or by genetic ablation, leads to exacerbated intestinal inflammation in the dextran sulphate sodium (DSS) colitis model [55, 56], which is typically associated with intestine epithelial barrier dysfunction and increased permeability to luminal agents that promote the inflammatory response [57]. In this model, IL-17A may exert its protective effects through facilitating tight-junction formation by inducing the expression of claudins in intestinal epithelial cells and by stimulating mucin production, thereby increasing mucosal barrier function [58]. Furthermore, when compared with wild-type mice, animals deficient in IL-22 show aggravation of the intestine inflammatory process following DSS administration, suggesting a tissue-protective role for this cytokine, that is produced by Th17 cells, which has been reported to stimulate intestinal epithelial cell proliferation and to enhance goblet cell function and mucus production, thus promoting intestinal barrier integrity [40, 59, 60]. Conversely, and opposed to the tissue-protective effects described for IL-17A and IL-22, it has been reported that IL-17F-deficient mice develop milder DSS-induced colitic symptoms than wild-type animals, thus suggesting that IL-17F exacerbates inflammation in this experimental model of colitis [56]. In fact, when DSS was administered to IL-17F knockout (KO) mice, a reduced chemokine mRNA colonic expression was observed compared with similarly treated wild-type controls [56]. At present, it is not known why IL-17A KO and IL-17F KO mice show different disease outcomes following DSS administration, even more when considering the fact that both cytokines bind the same receptor subunits IL-17RA and IL-17RC [34]; probably, the different ligand affinity, downstream signalling cascades, and receptor tissue distribution could clarify these differences. Considering all the above, Th17 cytokines have differential effects on the course and pathogenesis of IBD, and most probably, the final effect of these cytokines would depend on their relative abundance.

Moreover, recent studies have revealed the existence of an interaction between the Th17 and Th1 pathways in IBD. In fact, Annunziato et al. [15] demonstrated that some of the IL-17-producing T-cells found increased in CD mucosa also produce IFN-*γ*. Since then, different studies have corroborated the existence a functional plasticity of Th17 cells towards the Th1 cell linage. This process, although slightly controversial, seems to be responsible for the different biological functions ascribed to Th17 cells in intestine inflammatory conditions.

## 3. Th17 Cell Plasticity 

An essential approach by which the adaptive immune system orchestrates protective or tolerogenic responses to microorganisms relies on the differentiation of distinct functional subsets of CD4^+^ T cells from naive precursors, of a fixed, single antigenic specificity. This also ensures appropriate coordination between the innate and adaptive immune responses. In fact, it is well know that Th1 and Th2 cells control reciprocally their differentiation, since IL-12, IFN-*γ*, and the expression of T-bet inhibit Th2 differentiation, whereas IL-4 and GATA3 expressions antagonize Th1 polarization [7, 61]. This has been also reported, at least in murine models, for Th17 cells: both IFN-*γ* production by Th1 and IL-4 by Th2 cells exert inhibitory effects on IL-17A release by Th17 cells [62]. It is now established that once a CD4^+^ T cell has committed to either the Th1 or the Th2 lineages, conversion to the other lineage does not occur; in consequence, Th1 and Th2 cells develop into mature effector cells with rather stable phenotypes. Although this observation of a fixed phenotype and function was also initially applied to Th17 cells, research over the recent past years has ruled out this consideration.

### 3.1. Th1/Th17 Balance

**Figure 3:**
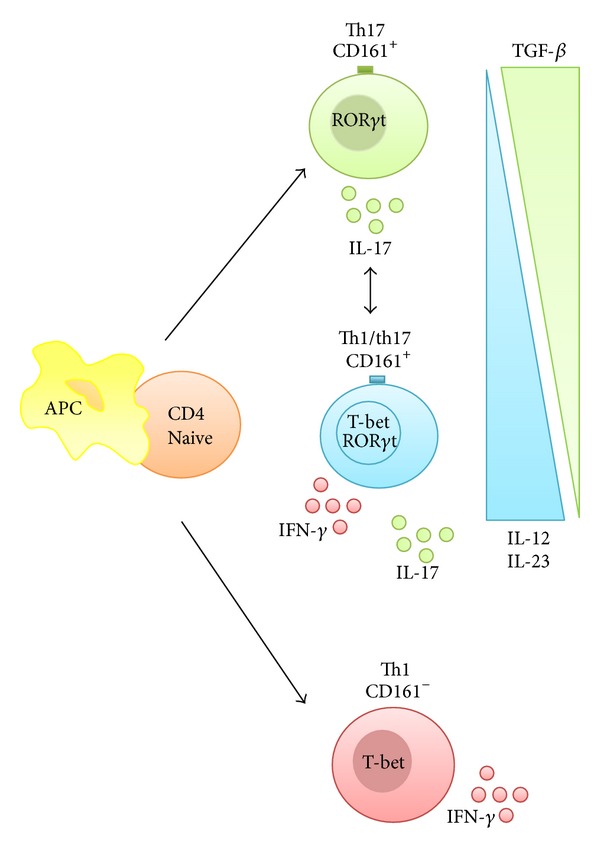
Th1/Th17 balance. Th17 cells exhibit plasticity towards the Th1 phenotype: Th17/Th1 cells or Th17-driven Th1 cells are cells capable of producing both IL-17 and IFN-*γ*. Th1 cells that derive from Th17 cells can be identified by the expression of CD161, a signature surface marker of Th17 cells progenitors, as opposed to classic CD161^−^ Th1 cells. TGF*β* is required for sustained expression of IL-17F and IL-17A, but in the absence of TGF*β*, both IL-23 and IL-12 downregulate ROR*γ*t and upregulate T-bet expression, which may result in the suppression of IL-17 and enhancement of IFN-*γ* production, in a STAT4- and T-bet-dependent manner.

As commented above, the existence of Th17 cells able to produce both IL-17 and IFN-*γ* was first reported by Annunziato et al. [15], who found them in the gut of patients with CD, thus suggesting that some of these Th17 cells could, at least in part, act like Th1 cells. As commented previously, Th17 cells required TGF*β* for sustained expression of IL-17F and IL-17A, but in the absence of TGF*β*, both IL-23 and IL-12 may act to suppress IL-17 and enhance IFN-*γ* production in a STAT4- and T-bet-dependent manner, albeit with distinct efficiencies [63].* In vitro* experiments supported these findings, since the incubation of Th17 cells with IL-12 induced either the production of IFN-*γ* and IL-17 (termed as Th17/Th1 cells) or IFN-*γ* instead of IL-17A (Th17-driven Th1 cells, or “nonclassic” Th1 cells) [15]. In fact, stimulation of these Th17 cells with IL-12 also enhanced the expression of the Th1-related transcription factor T-bet (and IFN-*γ*) and downregulated ROR*γ*t and IL-17 expression, thus indicating that IL-17-secreting T cells can be induced to differentiate into fully polarized Th1 cells [15] (Figure 3). Of note, whereas the* in vitro* assays reveal that IL-12 causes a rapid and an almost total suppression of the Th17 programme with the simultaneous upregulation of the Th1 transcription factor T-bet and IFN-*γ*, IL-23 requires several rounds of stimulation to cause a moderate deviation from a Th17 to a Th1 [63]. However, the role of IL-23 in promoting the switch from an IL-17A-producing T cell to an IFN-*γ*-secreting cells seems to be crucial* in vivo* [64].

It is interesting to note that the Th1 cells derived from Th17 cells express CD161, the surface marker of Th17 cells progenitors [17], as opposed to classic CD161^−^ Th1 cells, which are devoid of this marker. This shift of Th17 cells towards the Th1 phenotype has also been reported in murine experimental models, including IBD [63–66], and in other human conditions, including autoimmune polyarthritis [67] or oligoarticular juvenile idiopathic arthritis [68].

It has been proposed that the ability of Th17 cells to evolve into Th1 cells can be considered as a mechanism to prevent severe immunopathology, given the highly detrimental effects that can be derived from Th17 cell activity. All these findings would support the hypothesis that Th17-derived Th17/Th1 and Th1 cells, rather than Th17 cells alone, play a critical role in IBD [63, 64]. In consequence, those compounds interfering with both Th1 and Th17 cells' activity could be useful to facilitate the resolution of the ongoing mucosal inflammation in IBD.

### 3.2. Treg-Th17 Balance

**Figure 4:**
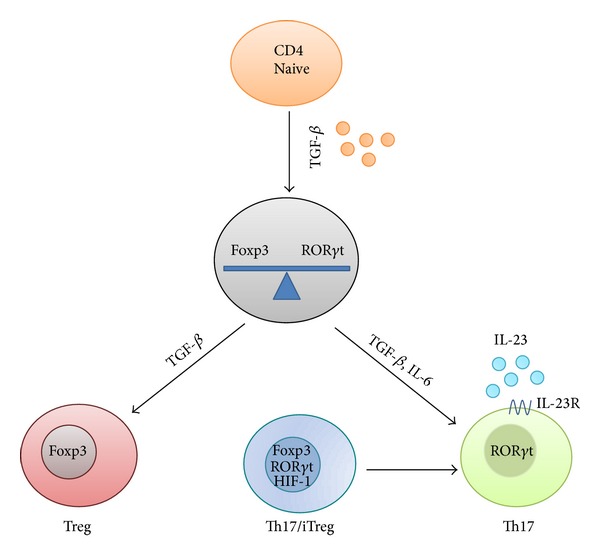
Treg/Th17 balance. Both Th17 and Treg cells control the proliferation of each other to maintain equilibrium. TGF*β* has a dual role in the differentiation of these two T-cell subsets. In naive T cells, TGF*β* promotes the development of Tregs by inducing Foxp3 expression; however, in the presence of IL-6, the production of Tregs is inhibited while the expression of IL-23R and ROR*γ*t is induced, thus promoting a Th17 phenotype. In addition, TGF*β*-induced Foxp3 expression negatively regulates Th17 cell differentiation by repressing IL-23R and ROR*γ*t expression. Actually, Th17/Treg precursors have been found, which express both ROR*γ*t and Foxp3 simultaneously and that can commit to one or other subsets under the control of factors such as HIF-1, which inhibits the differentiation toward the Treg lineage. This mutually dependent regulation of Th17/Treg differentiation is crucial to both immune homeostasis and pathogen clearance.

In addition to natural regulatory T cells (nTregs), which are of thymic origin, Tregs can be induced (iTregs) in the periphery under specific conditions. It has been reported that developmental pathways of Th17 and iTregs are closely related, being the differentiation of one another regulated to maintain their equilibrium, and thus influencing the outcome of immune responses in the context of inflammatory conditions, including IBD (Figure 4) [69]. It has been reported that Th17 cells reside mainly at barrier surfaces, particularly in the mucosa of the gut, where they protect the host from microorganisms that invade through the epithelium [23]. Similarly, iTreg cells are also mainly found in the intestinal mucosa, where they act to control excessive effector T-cell responses that might damage host tissues [70].

TGF*β* has been proposed as the critical common factor for Th17 and iTreg cells; thus, TGF*β* is able to promote the differentiation of naive T cells into regulatory cells in peripheral tissues, while in the presence of proinflammatory cytokines such as IL-6, it favors the differentiation of Th17 cells [23, 71]. Further* in vitro* studies have confirmed the relationship between these two T-cell subsets. TGF*β* upregulates ROR*γ*t expression [72], the central transcription factor for Th17 differentiation [26, 73, 74]. Similarly, TGF*β* induces the regulatory transcription factor Foxp3, which is crucial for the development of Tregs [75, 76]. However, it has been reported that the concurrent administration of IL-6 and TGF*β* to the culture media of naive T cells resulted in the inhibition of iTreg generation while the differentiation Th17 was promoted, a process that was associated with an increase in the expression of IL-23R and ROR*γ*t [23, 77]. On the contrary, in the absence of IL-23, the colonic Treg population is greater [78]. Supporting the reciprocal development of Th17 and Treg cells, it was reported that TGF*β*-induced Foxp3 expression represses IL-23R and ROR*γ*t expression, thus suggesting that the Treg phenotype regulates Th17 cell differentiation [69]. Similar conclusions were derived from studies of retinoic acid function, which induces Foxp3 and inhibits ROR*γ*t in Th17-inducing conditions, therefore promoting the development of Tregs [79, 80], and in STAT3 deficiency, a critical transcription factor related to Th17 development in humans [81, 82]. In fact, an increase in these suppressive cells might contribute to limiting the destructive Th effector responses. More recently, mouse studies have revealed the existence of dual Th17/Treg cell precursor, expressing both ROR*γ*t and Foxp3 simultaneously. The hypoxia-inducible factor-1 (HIF-1), a target gene of activated STAT3 [83], has been implicated in the regulation of Th17/Treg balance by promoting Th17 differentiation from these precursors [84]. One of the mechanisms proposed for this implies that HIF-1*α* inhibits Treg differentiation by targeting Foxp3 for ubiquitination and proteasomal degradation, thus stimulating Th17 differentiation in a STAT3-dependent fashion [84]. Furthermore, it has been described that the hypoxia-induced pathway can be itself activated by IL-17A and IL-17F, thus maintaining the HIF-1 pool and, as a result, perpetuating an existing Th17 response [85].

In consequence, it is interesting to note the important role that Th cell plasticity, driven by specific cytokines, can play in intestine conditions in which there is an altered immune response, like IBD. The active participation of TGF*β* in the development of two different T cell subsets is remarkable, that is, iTreg (anti-inflammatory) or Th17 (proinflammatory). In addition, the presence of the proinflammatory cytokine IL-6 in the intestine facilitates TGF*β*-induced Th17 differentiation instead of iTreg, thus providing a mechanism to combine immune homeostasis and pathogen clearance to extracellular bacteria at mucosal barrier locations, where a permanent interplay with intestinal microbiota occurs [86].

## 4. Intestinal Microbiota in IBD: Interaction with Th17 Cells

**Figure 5:**
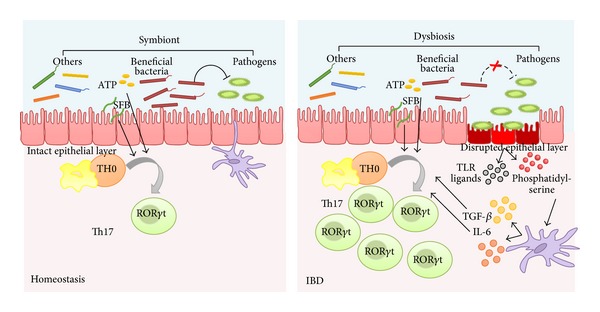
The intestine microbiota plays a key role in the pathogenesis of human IBD. Changes in the microbiota composition have been reported to be responsible for switching the tolerogenic response that normally occurs in healthy individuals to an activated, potentially pathogenic, immune response in IBD patients, characterized by the expansion of Th17 cells. Bacteria-infected apoptotic epithelial cells provide both TLR ligands and phosphatidylserine, which induce the production of IL-6 and TGF*β* from DCs, thus promoting Th17 differentiation.

The interaction of microbes with mucosal immune cells in the gastrointestinal tract seems to have a major role in priming and regulating immunity. In fact, different studies support the key role that intestine microbiota plays in the pathogenesis of human IBD (Figure 5). It has been reported that the intestinal mucosal damage that characterizes IBD is generated by an excessive or deregulated immune response against commensal microbes in the gut in individuals with a genetic susceptibility [87]. Increasing evidence also shows that there are substantial changes in the microbiota composition of IBD patients, leading to a situation termed as dysbiosis [88]. In this sense, Lepage et al. [89] have reported that in mucosal biopsy samples from UC patients there was a higher proportion of Actinobacteria and Proteobacteria and a lower proportion of Bacteroidetes. Similarly, a reduction of bacterial diversity has also been described in patients with CD [90]. Considering this, it has been proposed that these changes in the microbiota composition are responsible for switching the tolerogenic response that normally occurs in healthy individuals, to an activated, potentially pathogenic, immune response in IBD patients [91]. In the presence of “danger signals” triggered by microbe-associated molecular patterns, conditioned antigen-presenting cells might induce, through the secretion of different cytokines, the Th effector immune-inflammatory responses.

As commented before, Th17 cells are especially prominent in intestine mucosal surfaces, where they contribute, in cooperation with other T cells, to maintaining intestinal homeostasis and providing protection against invading microorganisms [92]. Strikingly, the endogenous microbiota seems to be involved in Th17 cell differentiation in the intestine under steady-state conditions [93, 94], being the presence of Th17 cells within different parts of the intestine reliant on the grade of colonization with commensal bacteria. Thus, in conventionally raised mice, Th17 cells are found at high numbers in ileum and colon lamina propria, but not in the duodenum, jejunum, mesenteric lymph nodes, or spleen [95, 96]. Supporting this, different studies have shown that the presence of Th17 cells in the intestine is noticeably reduced in antibiotic-treated or germ-free mice [72, 97]. In fact, when germ-free mice are subjected to fecal transplantation from control mice, an increased presence of Th17 cells in the gut lamina propria is evidenced within two weeks after colonization [72]. In particular, segmented filamentous bacteria (SFB), specific species of the Clostridia-related commensal bacteria, have been specially associated with the generation of Th17 cells [72, 93, 98]. Correspondingly, the inoculation of Gram-positive spore-forming bacteria to germ-free mice promotes the induction of IL-17- and IL-22-producing Th17 cells in the gut lamina propria [93]; furthermore, a direct correlation between the quantity of Gram-negative cytophaga-flavobacter-bacteroidetes and the presence of Th17 cells in the small intestine lamina propria has also been reported [72]. At present, the specific bacterial products involved in the homeostatic intestinal Th17 differentiation have not been fully elucidated, although several facts support flagellins as one of those: they are expressed by SFB, recognized by TLR5-expressing intestinal phagocytes, they induce IL-23 from CD103^+^ dendritic cells, and they are known to drive Th17 responses to enteric pathogens [99–103]. Microbiota-produced extracellular ATP can also be considered as an inducer of Th17 cells [97]. Likewise, indirect mechanisms may be involved, like IL-1*β* production induced by TLR recognition of intestinal bacteria by macrophages, since IL-1R signaling on intestinal T cells is required for homeostatic differentiation of Th17 cells [104]. How local production of IL-1*β* in the lamina propria directly affects naive T-cell differentiation within secondary lymphoid tissues remains to be clarified, but it is known that Peyer's patches are the location where naive CD4 T cells are closely associated with SFB and lamina propria macrophages [105].

It is remarkable that the homeostatic development of Th17 cells in response to commensal bacteria seems to be substantially different from the development of Th17 responses during inflammatory conditions; that is, Th17 cells highly infiltrate the inflamed areas of the intestine in mice subjected to experimental colitis [99, 103]. Different studies have confirmed the contribution of commensal gut microbiota to Th17 cell expansion in IBD. In this sense, it has been reported that bacteria-infected apoptotic IECs provide both TLR ligands and phosphatidylserine, which promote the production of IL-6 and TGF*β* from DCs, thus facilitating Th17 differentiation [106]. Supporting this, Elson et al. [107] demonstrated that the transfer of cecal bacterial antigen-specific C3H/HeJBir (C3Bir) CD4 (+) T-cell line to C3H/HeSnJ SCID mice induced a colitic process associated with enhanced production of Th17-derived cytokines, which is more severe than that caused by the transfer of Th1 cells.

New insights into Th17 cell involvement in inflammatory or infectious diseases have recently been derived from the generation of an IL-17A-eYFP reporter mouse. Using this reporter mouse, Hirota et al. have demonstrated that IFN-*γ*-producing CD4^+^ T cells are actually IL-17A producers, as identified by their expression of eYFP [64]. Moreover, they have shown that the production of other proinflammatory cytokines such as GM-CSF, IL-2, and TNF-*α* by effector CD4^+^ T cells is derived almost exclusively from “ex-Th17” cells, with no apparent contribution of Th1 cells [64]. All these findings indicate that Th17 cell fate is shaped by the* in vivo* microenvironment, in which chronic inflammatory states promote phenotype switching and the expression of IFN-*γ* (and other proinflammatory cytokines) by Th17 cells. It is also evident that the ability of the intestinal microbiota to strongly modulate the induction of Th17 responses can influence the susceptibility to intestinal inflammation [108, 109].

## 5. Novel Th17-Targeted Therapies in Intestine Inflammation

Given the key role attributed to Th17 cells in intestine inflammation, they have been proposed as a feasible therapeutic target to control intestinal inflammatory conditions. Theoretically, potential strategies would include the blockade of Th17 cell differentiation and expansion, the neutralization of the cytokines produced by these cells, and the inhibition of the specific transcription factors required for Th17 cell function. Studies evaluating these possibilities have been performed on experimental models of mouse colitis, revealing in some cases conflicting results.

### 5.1. Blocking the Differentiation and Amplification of Th17 Cells

Since different innate immune cells, like DC and macrophages, and their inflammatory cytokines, such as IL-1*β*, IL-6, IL-21, and IL-23, have a key role in promoting the differentiation and proliferation of Th17 cells, the use of neutralizing monoclonal antibodies for these cytokines or the antagonism of their receptors may well be a reasonable strategy for the treatment of IBD.

In this sense, Fina et al. [39] have demonstrated that the blockade of IL-21R by the administration of IL-21R/Fc attenuates inflammation in mice with established DSS colitis. The same authors have observed that the neutralization of IL-21 by an anti-IL-21 antibody reduces IL-17 secretion by lamina propria lymphocytes isolated from IBD patients [39]. Similarly, the administration of a neutralizing IL-21 antibody to DSS-colitis mice decreased the colonic T-cell infiltrate and the production of IL-6 and IL-17A in the inflamed colonic tissue [110].

Monoclonal antibodies directed against interleukin-12/23 p40 have also shown efficacy in murine colitis models [111, 112]. More recently, the human monoclonal antibody against IL-12 and IL-23, ustekinumab, has shown effectiveness in patients with moderate-to-severe CD, especially in those in which anti-TNF therapy had previously failed [113]. The success of ustekinumab might be due to its ability to simultaneously inhibit Th1 and Th17 cells; both of them are clearly implicated in IBD pathogenesis.

### 5.2. Inhibition and/or Neutralization of Th17 Cytokines

Using an acute model of trinitrobenzenesulfonic acid- (TNBS-) induced colitis, Zhang et al. [114] have demonstrated that IL-17R knockout mice are significantly protected against colonic inflammation, even though they have equivalent amounts of local IL-23 and higher levels of IL-12p70 and IFN*γ* in comparison to wild-type mice. Moreover, in this study, these authors also stated that the overexpression of an IL-17R IgG1 fusion protein significantly attenuated colonic inflammation after acute TNBS-colitis induction [114]. However, contrasting results have been obtained after the inhibition of the function of IL-17 in animal models of IBD, probably due to the different functions exerted by IL-17A and IL-17F in the specific context of intestinal inflammation. Whereas murine DSS-induced colitis was worsened in IL-17A KO mice, a clear improvement of the colonic inflammatory process was observed in IL-17F KO mice [56]. The results obtained in humans regarding IL-17 inhibition are also controversial, since secukinumab, a human anti-IL-17A antibody, failed to show any beneficial effect in CD patients [115]. For this reason, current studies are now focused on evaluating combined IL-17A and IL-17F blockade as a potential strategy for IBD therapy [116].

The inhibition of IL-17 production by T cells has also been given some attention recently. In this context, vidofludimus, a novel oral immunomodulatory drug that inhibits dihydroorotate dehydrogenase and lymphocyte proliferation* in vitro*, has been demonstrated to reduce the expression of IL-17A and IL-17F, as well as IFN-*γ*, independently of its effects on lymphocyte proliferation, through the inhibition of STAT3 and nuclear factor-*κ*B activation [117]. In addition, recent clinical trials have shown the efficacy, safety, and tolerability of vidofludimus in IBD patients [118].

When considering Th17 cells plasticity, it has been described that when Th17 cells lose their ability to secrete IL-17A and turn into IFN-*γ* producers, they express high levels of aryl hydrocarbon receptor (AhR) [119, 120], suggesting that AhR might be controlling the activity of these cells. AhR is a transcription factor ubiquitously expressed in vertebrate cells, which mediates a range of cellular events in response to different ligands including derivatives of tryptophan, such as 6-formylindolo(3, 2-b)carbazole (Ficz) [121]. Activation of AhR results in enhanced production of IL-22, together with a reduction of Th1 and Th2 cytokines [119, 122, 123]. Monteleone et al. [124] have shown that Ficz administration to colitis mice ameliorates colonic inflammation, an effect associated with an inhibition of Th1 cytokines and upregulation of IL-22. This beneficial effect was reversed after blockade of IL-22 with a neutralizing antibody, thus indicating that induction of IL-22 is one of the major mechanisms by which AhR signals control pathogenic responses in the gut. In fact, AhR expression is markedly downregulated in the inflamed tissue of CD patients [124], and, in agreement to what observed in mice, treatment of human IBD mucosal cells with Ficz resulted in decreased IFN-*γ* expression and upregulation of IL-22.

### 5.3. Inhibition of Th17-Cell-Specific Transcription Factors

Possible pharmacological targets in this regard comprise mainly the transcription factors ROR*γ*t and STAT3, which control TH17 polarization and function. In this sense, pioglitazone, a nuclear receptor peroxisome proliferator-activated receptor gamma (PPAR-*γ*) agonist, has been shown to inhibit human and murine Th17 differentiation by reducing TGF*β*/IL-6-induced expression of ROR*γ*t [125], and indeed, it has proven to be favourable in mouse DSS-induced colitis [126]. More recently, it has been proposed that the beneficial effects exerted by mesenchymal stem cells in experimental colitis can be associated with a downregulation of both Th1-Th17-driven autoimmune and inflammatory responses derived from the inhibition of ROR*γ*t activity together with the activation of CD4(+)CD25(+)Foxp3(+)Treg cells [127]. Finally, the recently described synthetic triterpenoids, dual small-molecule inhibitors of NF-*κ*B and STAT3, may as well have potential benefits in intestinal inflammation [128].

All in all, further randomized clinical trials are required to confirm the benefits of targeting Th17 cells in IBD patients.

## 6. Concluding Remarks

Being the immune system the main mediator of tissue injury in IBD, it represents one of the key targets for future therapeutic interventions aimed at controlling this chronic and debilitating disease. New cellular components and features of the immune system have been discovered in the past decades that have revolutionized our understanding of the mechanisms underlying the development of IBD, that is, the case of Th17 cells, which hold a key position in IBD immunopathogenesis, especially due to their location and their remarkable ability to adapt to changing environmental conditions. It is now acknowledged that Th17 cells exhibit a certain degree of plasticity, which accounts for their key role in preserving homeostasis, especially in barrier tissues. This is particularly relevant in the gastrointestinal tract, where the immune system has to carefully balance the sustainability of a needed commensal microbiota, while preventing the invasion of host tissues. Although much progress has been made in revealing the role of Th17 cells during intestinal inflammation, there is still much to be learned. Targeting Th17 cells in an attempt to control other autoimmune diseases is already showing promising results. Therefore, a better understanding of this T-cell subset could lead to solid foundations for the development of novel effective biological agents for IBD management.
